# *Thymus ad astra*, or spaceflight-induced thymic involution

**DOI:** 10.3389/fimmu.2024.1534444

**Published:** 2025-01-24

**Authors:** Wataru Muramatsu, Maria Maryanovich, Taishin Akiyama, George S. Karagiannis

**Affiliations:** ^1^ Laboratory of Immune Homeostasis, RIKEN Center for Integrative Medical Sciences, Yokohama, Japan; ^2^ Immunobiology, Graduate School of Medical Life Science, Yokohama City University, Yokohama, Japan; ^3^ Department of Cell Biology, Albert Einstein College of Medicine, Bronx, NY, United States; ^4^ Ruth L. and David S. Gottesman Institute for Stem Cell and Regenerative Medicine, Albert Einstein College of Medicine, Bronx, NY, United States; ^5^ Cancer Dormancy Institute, Montefiore-Einstein Comprehensive Cancer Center, Bronx, NY, United States; ^6^ Department of Microbiology and Immunology, Albert Einstein College of Medicine, Bronx, NY, United States; ^7^ Tumor Microenvironment Program, Montefiore-Einstein Comprehensive Cancer Center, Bronx, NY, United States; ^8^ Gruss-Lipper Biophotonics Center, Albert Einstein College of Medicine, Bronx, NY, United States; ^9^ Integrated Imaging Program for Cancer Research, Albert Einstein College of Medicine, Bronx, NY, United States; ^10^ The Marilyn and Stanely M. Katz Institute for Immunotherapy for Cancer and Inflammatory Disorders, Montefiore-Einstein Comprehensive Cancer Center, Bronx, NY, United States

**Keywords:** thymus, spaceflight, involution, cosmic radiation, microgravity (μg), circadian rhythms, psychosocial stress

## Abstract

Spaceflight imposes a constellation of physiological challenges—cosmic radiation, microgravity, disrupted circadian rhythms, and psychosocial stress—that critically compromise astronaut health. Among the most vulnerable organs is the thymus, a cornerstone of immune system functionality, tasked with generating naive T cells essential for adaptive immunity. The thymus is particularly sensitive to spaceflight conditions, as its role in maintaining immune homeostasis is tightly regulated by a balance of systemic and local factors easily disrupted in space. Cosmic radiation, an omnipresent hazard beyond Earth’s magnetosphere, accelerates DNA damage and cellular senescence in thymic epithelial cells, impairing thymopoiesis and increasing the risk of immune dysregulation. Microgravity and circadian rhythm disruption exacerbate this by altering immune cell migration patterns and stromal support, critical for T-cell development. Psychosocial stressors, including prolonged isolation and mission-induced anxiety, further compound thymic atrophy by elevating systemic glucocorticoid levels. Ground-based analogs simulating cosmic radiation and microgravity have been instrumental in elucidating mechanisms of thymic involution and its downstream effects on immunity. These models reveal that long-duration missions result in diminished naive T-cell output, leaving astronauts vulnerable to infections and possibly at high risk for developing neoplasia. Advances in countermeasures, such as pharmacological interventions targeting thymic regeneration and bioengineering approaches to protect thymic architecture, are emerging as vital strategies to preserve immune resilience during prolonged space exploration. Focusing on the thymus as a central hub of immune vulnerability underscores its pivotal role in spaceflight-induced health risks. Understanding these dynamics will not only enhance the safety of human space missions but also provide critical insights into thymus biology under extreme conditions.

## 1 Overview of spaceflight stressors affecting the immune system

Astronauts experience hostile environmental changes and stressors during spaceflight, broadly classified into four distinct categories: cosmic radiation, microgravity, circadian derailment, and psychosocial stressors, the latter including social isolation, various constraints and fears, crew member conflicts, and extreme pressure for exceptional mission performance. Together, these factors have a significant impact on many physiological systems in the body, eventually posing an obstacle to long-term space missions (1–11).

Beyond the Earth’s protective magnetosphere, astronauts are exposed to elevated levels of galactic cosmic radiation (GCR) and solar energetic particles (SEP), both of which pose significant health risks. GCR, consisting of high-energy protons and atomic nuclei, and SEP, primarily composed of charged particles from the Sun, are highly penetrating, and can damage cellular structures, DNA, and tissues. Due to the challenges in replicating the precise radiation environment of space in Earth-based facilities, it remains difficult to fully assess the long-term health consequences of chronic exposure to GCR and SEP (12), estimated to be approximately 1mSV per day spent at the international space station (ISS) (13). Nevertheless, the primary risks associated with this exposure include an increased likelihood of cancer development, central nervous system (CNS) defects that contribute to cognitive and behavioral impairments, as well as neurological and cardiovascular disorders. Additionally, radiation exposure has been shown to lead to an acute or progressive decline in immune system functions, which can severely impact astronaut health and mission success (12).

In addition to the constant but low-dose GCR/SEP exposure, any gravitational forces less than 1x10^-3^ g, including those at ~1x10^-6^ g (i.e., microgravity), which are typically experienced during spaceflight, may impose additional stress, particularly to the musculoskeletal system (14–18). Indeed, during international space station (ISS) missions, astronauts experience a significant reduction in bone mineral density along with muscular atrophy, triggering the inclusion of physical training routines during spaceflight as an essential countermeasure (19–21). However, it has long been established that muscles and bones related to posture and weight are inherently linked to the gravitational load, and as such, can be severely affected by its perturbations (22–24). On the other side, the effect of microgravity on other organ systems, especially the immune system, are not appreciated, and key observations are only now beginning to emerge (25).

Spaceflight also presents unique challenges to circadian rhythms, primarily due to absence of a consistent 24-hour light-dark cycle. In spaceflight, the continuous artificial lighting and the lack of natural sunlight cues disturb the body’s internal clock, leading to fragmented sleep patterns and impaired performance (26–30). Such disruptions pose significant risks for long-term missions, as sleep disorders and desynchronized circadian rhythms can heighten the behavioral risks and psychiatric disorders (31). In addition, a number of studies over the years highlight a complex link between circadian rhythms and immune function (32–34), which may lead to severe symptoms, such as obesity, metabolic syndromes, cardiovascular disease, and cancer (31, 35–37), thus inferring that such disruptions may further impact health and resilience in space (31).

Psychosocial stressors in spaceflight, including isolation, confinement, and interpersonal challenges, can significantly impact astronaut health, including feelings of loneliness, anxiety, and depression, exacerbated by the absence of natural light, radiation effects, microgravity effects, and/or long-duration separation from family. Observational research from ISS missions, the Mars500 simulation, and other space-analogue environments, has documented significant psychological strain, mood swing, irritability, cognitive impacts, and interpersonal conflicts (38–43). Currently, there is ample evidence that psychosocial stressors can lead to the dysfunction of the immune system (44), although establishing direct causative link is challenging, due to the complex interplay of factors unique to spaceflight conditions.

In summary, the stressors encountered during spaceflight—cosmic radiation, microgravity, circadian disruption, and psychosocial challenges—can independently affect astronaut health, particularly the immune system. These factors likely interact with each other, amplifying their negative impact on immune functions. Understanding the mechanisms behind these interactions is therefore crucial for mitigating health risks on long-duration missions. A compromised immune system can hinder the ability to fight infections and recover from illness aboard a manned mission, making it essential to safeguard immune health for the success of space exploration.

## 2 Effects of spaceflight stressors on thymus homeostasis and involution

Several studies have shown that spaceflight stressors perturb immune system homeostasis and immunological responses to pathogens (45–58). The human alpha herpesviruses, such as herpes simplex virus (HSV-1) and varicella zoster virus (VZV), may enter a latent state in cranial nerve ganglia but can reactivate when stress impacts immune regulation (59, 60). During spaceflight, reactivation of viruses like Epstein-Barr (EBV), VZV, HSV-1, and cytomegalovirus (CMV) often occurs without symptoms, although live virus particles have been isolated, and viral shedding rates increase with mission length. As a consequence, extended missions (>180 days) could heighten the risk of developing symptomatic infections in astronauts, such as skin rash dermatitis, posing an incremental health concern and impairing their performance (61–67). Additional evidence of adverse immunological manifestations occurring during either short- or long-duration spaceflight missions comes from measured disturbances in immune-related cytokine levels in astronauts, such as tumor necrosis factor-α (TNFα) and interferon-γ (IFNγ) among others (58, 68, 69), which are also known to be essential for thymus homeostasis (70–72).

Studies on the impact of spaceflight on immune system development and lymphoid organ homeostasis are limited, and comprise of mixed observations from astronauts and rodents (73–75). With regards to the bone marrow, it has been demonstrated that spaceflight may disrupt both the mesenchymal (MSC) and hematopoietic stem cell (HSC) compartments, thus affecting the differentiation and maturation of descendant lineages, particularly B cells, myeloid cells, and erythrocytes (15, 20, 76, 77). An in-depth analysis of the impact of spaceflight on bone marrow homeostasis, however, is beyond the scope of this article, but the readers are encouraged to review relevant work by others (25, 78–81).

Concrete evidence that spaceflight affects thymic functions and causes involution has been recently demonstrated in a critical study that investigated the effects of long-term spaceflight in 16 astronauts during a median 184-day mission aboard the International Space Station (ISS) (82). Thymopoiesis was assessed in each astronaut at multiple timepoints by measuring T-cell receptor excision circles (TREC) (82), a molecular marker detectable in recent thymic emigrants (83–85). Samples were collected approximately 180 days before launch, within 2–4 hours of landing, and up to 180 days post-landing. A consistent and significant decline in thymopoiesis was observed immediately after landing, followed by a return to preflight levels within days to weeks, eventually stabilizing to the preflight range (82). Interestingly, the study identified an inverse correlation between cortisol levels and thymic output (82), suggesting that glucocorticoid-induced thymocyte apoptosis may in part contribute to reduced thymopoiesis during spaceflight. Thymic involution was also observed in experimental mice housed aboard the ISS for 35 days (86), and the Space Shuttle Atlantis for 13 days (87). Notably, significant thymic mass loss occurred only in the former, although DNA fragmentation assays indicated increased apoptosis in the thyme of mice exposed to spaceflight in the latter (86, 87). Therefore, mission duration is critical for a substantial impact on thymic integrity. Altogether, these findings suggest that extended space missions compromise immune and thymic function, and increase infection susceptibility. Additionally, they underscore not only the critical need for developing countermeasures to enhance immune resilience, but also the importance of developing faithful ground-based analogues to investigate in more detail immune dysfunctions from a more mechanistic perspective.

## 3 Ground-based models that recapitulate spaceflight-induced thymic involution

Given the high cost and limited opportunities to conduct spaceflight experiments with model organisms, ground-based models simulating spaceflight conditions have been developed as practical and accessible alternatives. These models aim to replicate key stressors encountered during spaceflight, as outlined above, offering insights into their physiological effects on thymus homeostasis. Studying the impact of spaceflight on immune system development is particularly challenging in humans due to ethical and logistical constraints. As a result, rodent models have become the primary choice for such investigations, providing valuable data, while serving as an approximation of human responses. In this section, we will thus describe and critically assess the most well-established ground-based models currently regarded as relatively equivalent to actual spaceflight conditions, highlighting their utility, limitations, and relevance to understanding spaceflight-induced stressors.

### 3.1 Cosmic radiation

Most studies investigating the impact of space radiation on the hematopoietic system have been conducted using monoenergetic electron and gamma-ray beams. Exposure of rats to gamma rays was performed on board of the satellite Cosmos-690 along with a control group receiving matched dosing on Earth (88). Hematopoietic assessments demonstrated a significantly enhanced effect in rats irradiated in spaceflight, when compared to rats irradiated on Earth, with severe suppression of bone marrow hematopoiesis and thymopoiesis (88). Along these lines, the exposure of rat thymocyte suspensions to Co-60 gamma-rays induced severe apoptosis and distinct morphological and functional changes in thymocytes, assessed via electron microscopy, DNA fragmentation assays, and biochemical assays (89). Further mechanistic insights revealed an activation of intracellular and intranuclear proteases, typical of the extrinsic apoptotic pathway, leading to the degradation of mitochondria and the release of pro-apoptotic factors (90). However, a similar study on Cosmos-690 that measured the combined effects of microgravity and ionizing radiation from a Cs-137 source did not reveal significant changes in thymus weight and spleen after irradiation, although bone marrow hematopoiesis was affected (91). In two separate studies, exposure to Fe-56 particles, or C-12 (6+) ions induced severe spleen and bone marrow defects, as well as thymic involution in adult female C57BL/6 or King-Ming strain mice, respectively, demonstrating varying degrees of susceptibility for lymphocyte populations (92, 93). Collectively, these studies demonstrate the pleiotropic effects of diverse monoenergetic ion sources on thymic structure and function, although the precise mechanistic insights behind the observed variability are not fully understood. It should be noted however, that most research to evaluate health risks from space radiation has been historically performed via acute exposure to such monoenergetic single-ion beams, as outlined in the studies above. Nevertheless, it has now been established that such exposures do not faithfully recapitulate the intricacies of the galactic ray environment in our solar system (12), and as such, should be interpreted with caution.

To address such concerns, ground-based GCR simulators have been developed to expose experimental animals and cell cultures to “mission-relevant” radiation doses. These simulators incorporate diverse vehicle and shielding configurations, high design fidelity, precise material characterization, mission duration considerations, and realistic solar conditions (94–101). The most advanced, developed by the NASA Space Radiation Laboratory (NSRL) at Brookhaven National Laboratory, delivers radiation doses comprising a mixture of protons (~65%-75%), helium ions (~10%-20%), and heavier ions (C, O, Si, Ti, Fe) (102). To more closely replicate the low-dose rates found in space, this system can additionally fractionate sequential field exposures over daily intervals for 2 to 6 weeks, allowing state-of-the-art cellular and animal model systems to be exposed to mission-relevant radiation (12). So far, sophisticated GCR simulation has been used to examine various organ system adaptations to space- and mission-relevant radiation doses, including the gastrointestinal, endocrine, cardiovascular, immune, ocular, and central nervous systems (103–111). To our knowledge however, there are currently no studies explicitly dedicated to evaluating thymus architecture and functions using GCR simulators.

Instead, most studies using GCR simulators have focused on the effects of mission-relevant GCR exposure on the bone marrow. For instance, mice exposed to mission-equivalent GCR doses showed increased osteoclast activity and trabecular bone loss, suggesting alterations in the endosteal niche (112, 113), which regulates hematopoiesis (114–116). Simulated SEP and GCR radiation also disrupted the ability of MSCs to support hematopoiesis and directly impaired human hematopoietic stem cell (HSC) functionality, inducing DNA damage and mutations. Sequential exposure to protons and iron ions, mimicking deep space radiation, was particularly harmful to HSC genome integrity. Notably, sequential exposure to protons and iron ions—mimicking the complexity of deep space radiation—proved significantly more harmful to HSC genome integrity and function than exposure to either particle type alone (117). These findings emphasize once again the importance of simulating the full spectrum of galactic cosmic radiation for accurate assessments. Collectively, these studies suggest that GCR may impact thymopoiesis indirectly by disrupting bone marrow hematopoiesis and the influx of early thymic progenitors. However, the possibility of direct effects of GCR/SEP on the thymus itself cannot be excluded, as indicated from astronaut observations and rodent experiments (25, 82, 118).

### 3.2 Microgravity

Development of ground-based models replicating microgravity is particularly challenging. Parabolic flights conducted on Earth on one side accurately replicate the gravitational conditions experienced in orbital spaceflight. Indeed, numerous experiments have explored the effects of microgravity on physiological systems using this approach (119). However, the duration of induced microgravity during parabolic flights is typically limited to several minutes, making it unsuitable for assessing long-term effects. Since the impact of microgravity on the thymus likely occurs over days, this model is likely inadequate for evaluating thymic responses.

For *in vitro* experiments, devices such as the clinostat and magnetic levitation are commonly used to simulate microgravity. These models are primarily restricted to cultured cell studies, thus posing an impediment to recapitulate the complex microenvironment of lymphoid organs. However, fetal thymic organ cultures, typically derived from E14 to E16 mouse embryos, can be maintained *in vitro*, and physiologically recapitulate stromal-thymocyte interactions (120), potentially enabling the study of simulated microgravity effects on thymocyte development. Indeed, a clinostat study demonstrated a reduction in CD4^+^CD8^+^ thymocytes after a 12-day fetal thymic organ culture (121). Nonetheless, these findings should be interpreted with caution, as the cellular composition of the fetal thymus differs significantly from the adult one, highlighting the need for further studies to understand the effects of microgravity on adult thymic function.

The hindlimb unloading (HU) model, also known as the tail suspension model, is frequently used to simulate weightlessness in rodents (122). This model removes weight-bearing from the hindlimbs, impacting the musculoskeletal system, and causing a redistribution of body fluids towards the head, analogous to the fluid shifts observed in humans under microgravity conditions. Besides musculoskeletal ramifications, short-term (2-day) HU in mice resulted in reduced thymic mass, with CD4^+^CD8^+^ thymocytes being particularly sensitive (123). The total number of mature single-positive (CD4^+^CD8^-^ or CD4^-^CD8^+^) thymocytes was markedly reduced and accompanying TUNEL assays indicated an increase in apoptotic cells in the thymus (123). Combined with steroid receptor blocking experiments, these findings also suggested that corticosterone-dependent apoptosis is responsible for thymic cell reduction during short-term HU. Another study revealed that osteopontin is involved in HU-induced thymic apoptosis by regulating corticosterone levels during a 3-day HU (124). Subsequent studies have demonstrated that circulatory osteopontin can interfere with the hypothalamus-pituitary-adrenal (HPA) axis, thus regulating steroid hormone production and modulating stress responses (125), although the precise mechanisms behind this regulation have not been elucidated. In contrast, long-term HU does not selectively reduce CD4^+^CD8^+^ thymocytes, despite a decrease in overall thymic mass (126). Instead, long-term HU led to significant decline in medullary thymic epithelial cells (TECs), particularly those expressing high levels of CD80 and the autoimmune regulator (AIRE). Consistent with this, the expression of tissue-specific antigens was downregulated in the thymus of long-term HU-loaded mice (126). Together, these findings indicate that the effects of HU are distinctly time-dependent: short-term HU selectively induces corticosterone-driven apoptosis in CD4^+^CD8^+^ thymocytes, while long-term HU impacts all thymocytes in a non-selective manner, along with the AIRE^+^ mTEC (i.e., mTEC^hi^) population, likely through apoptosis-independent mechanisms, despite the persistently elevated corticosterone levels in either condition.

Several studies have indicated that spaceflight induces thymic involution in mice with similar lesions to those observed in the HU model (74, 75, 86, 87, 118, 126), suggesting that hindlimb unloading may effectively replicate some aspects of spaceflight conditions. However, a key limitation of the HU model is that it not only simulates weightlessness, but also induces psychological stress in mice, which can act as a model for depression (127). This introduces complexity in interpreting results, because it becomes challenging to differentiate whether thymic atrophy is due to stress, musculoskeletal changes, fluid redistribution, or a combination of these factors. All these conditions are present under both microgravity and hindlimb unloading environments.

### 3.3 Circadian derailment

Circadian rhythms control many aspects of human physiology, affecting daily variations in body temperature, blood pressure, and hormone levels and coordinate function across different organ systems, including neurological, metabolic, endocrine, cardiovascular, and immune (128). Circadian rhythmicity in the body is entrained by photic cues and a tight network of central and peripheral clocks enabled by a neural pacemaker directly responsive to environmental and behavioral states such as the sleep-wake cycles, feeding, metabolic cues, and secretion of hormones (particularly glucocorticoids) (129–131).

Circadian derailment is considered a risk factor during space missions by NASA. During space flight, astronauts are exposed to changes in microgravity, which impose pathophysiological effects on circadian rhythmicity, leading to derailment as a consequence of disturbed sleep, wakefulness, and feeding patterns (30, 31). Astronauts working at the ISS experience 16 sunrises and sunsets within a 24-hour period, impairing the 24-hour diurnal cycle experienced on earth. Even more so, the profound workload during space missions, which requires astronauts to complete highly complex tasks during long periods of time, contributes to the disruption of sleep-wakefulness cycles that collectively affect the body’s physiological diurnal rhythms (132–135). Derailment of circadian rhythm affects human health as increased occurrence of cardiovascular disease (CVD) (136), metabolic disorders (137), and cancer (138–140) were reported to be associated with shift work or frequent time zone travel. Coupled with other hazards of spaceflight, derailment of circadian oscillations during space missions may result in considerable risk to astronaut health, including not only sleep deprivation and diminished alertness, loss of cognitive abilities, depression, and anxiety (141, 142), but also the development of metabolic syndrome, CVD and cancer.

The hematopoietic and immune systems are particularly sensitive to circadian derailment. Mobilization and trafficking of leukocytes and hematopoietic stem and progenitor cells (HSPCs) between lymphoid organs and other tissues in the body is tightly regulated by central and peripheral clocks (34, 143–145). Innate immune cells (including granulocytes, monocytes, and macrophages) and T and B cells exhibit strong circadian oscillations in peripheral blood, peaking during the behavioral rest phase (daytime in rodents, and at night in humans) (146–148). Oscillation of blood lymphocytes was demonstrated to depend on glucocorticoids, catecholamines, and hypoxia-inducible factor 1a (HIF-1a) (149–151) that mediate rhythmic expression of chemokine receptors (e.g., CXCR4, CXCR5, CCR7, CX_3_CR1) that oscillate in phase with tissue-specific chemokines (e.g., CXCL12 in bone marrow and lung and CCL21 in lymph nodes) and endothelial adhesion molecules, including P and E-Selectin, Intercellular adhesion molecule-1 (ICAM-1), ICAM-2, and vascular cell adhesion molecule-1 (VCAM-1) across lymphoid and other organs, (including liver, skin, gut and lung) (146). Besides leukocyte trafficking and recruitment into tissues, recent studies have demonstrated that innate and adaptive immune responses depend on circadian rhythmicity, including response to pathogens, B cell development, and T cell differentiation. Circadian control of immune response is not the scope of this review; a detailed summary of this topic can be found elsewhere (34, 152).

Whether and how derailment of circadian rhythmicity affects thymic function and T cell development is less known. Although it has been shown that loss of intrinsic circadian rhythms by deletion of the master clock regulator Brain and Muscle Arnt-like protein-1 (Bmal1*)* in thymocytes does not affect T cell development (153), CD4^+^ single positive (SP4) thymocyte emigration from the thymus was shown to be regulated by circadian rhythms, as well as rhythmic expression of emigration related-molecules sphingosine 1-phosphate receptor (S1PR1) and C-C chemokine receptor 2 (CCR2) (154). As spaceflight and altered microgravity were shown to induce thymic involution (75, 87), it is yet to be determined to what extent the derailment of circadian rhythmicity contributes to thymic dysfunction. Future studies utilizing ground-based models of acute and chronic jet lag will directly test this question and determine how circadian derailment affects thymic structure and functionality. However, as spaceflight is associated with microgravity disruption, which can also contribute to impaired circadian rhythmicity (155–158), a combination of jet lag with hindlimb unloading may be necessary to properly simulate spaceflight conditions that derail circadian rhythmicity. Furthermore, it will be important to show to what extent derailment of circadian rhythmicity during spaceflight contributes to the development of CVD and cancer, as defective immune response contributes to the pathogenicity of either condition.

### 3.4 Psychosocial stress

During prolonged space missions, astronauts are exposed to extreme environments for extended durations, potentially leading to adverse physical and mental health effects, such as depression and cognitive impairment. The concept of “long-term spaceflight composite stress” (LSCS) encapsulates the multifaceted sources of stress encountered in space (142). Among these, psychosocial stress stands out as a significant contributor, distinct from well-known hazards like cosmic radiation, microgravity, and circadian disruptions (142). Instead, it arises from factors such as social isolation, confinement in cramped and crowded spaces, cultural differences and conflicts among crew members, homesickness, performance anxiety, and persistent noise from the onboard equipment (e.g., fans, exercise machines, life-support systems) (142). However, studying the isolated effects of psychosocial stressors on normal physiology, and the immune system, in ground-based rodent models presents significant challenges. The multifactorial nature of these stressors is inherently difficult to replicate in controlled laboratory settings (159–164). Additionally, fundamental differences between humans and rodents further complicate such models: the human brain, with its unparalleled complexity and advanced cognitive and emotional capacities, processes psychosocial stimuli in ways that are not easily mirrored in rodent counterparts (165).

The effects of LSCS have been previously studied, although psycho-social factors have not been isolated from other spaceflight-associated stressors. For example, a 42-day simulation combining microgravity, isolation, noise, circadian rhythm disturbances, and low pressure demonstrated significant weight loss, anxiety, memory deficits, and depression in rats. These behavioral changes correlated with reduced postsynaptic density thickness and synaptic interface curvature, indicating impaired synaptic plasticity in the hippocampus of LSCS-exposed rats (166–168). While a connection between depression and immune system dysregulation is loosely supported (169), direct evidence linking LSCS to immunological and thymic functions is still lacking.

## 4 Conclusions, future perspectives, and translational (space-)blocks

Spaceflight-induced thymic involution is a complex phenomenon influenced by composite stressors, highlighting the necessity of developing faithful ground-based models to complement spaceflight research. The logistical and financial challenges of conducting rodent experiments in space make such models indispensable. However, most existing models focus on isolating single stressors, such as hindlimb unloading to simulate microgravity, or galactic cosmic ray (GCR) simulators to replicate radiation exposure. While these approaches provide valuable insights into the individual contributions of specific stressors to thymic dysfunction, they fail to replicate the multifactorial nature of the space environment, where these stressors act simultaneously. Multi-hit models, also known as long-term spaceflight composite stress (LSCS) models, which incorporate multiple stressors on the other side may offer a more comprehensive solution to this challenge, as they may reveal their synergistic or additive effects (73, 111, 142, 166, 170). Notably, certain ground-based models, such as the HU may be inherently multifactorial themselves, raising further concerns regarding their interpretation. As mentioned above HU introduces psychological stress to mice (127), thus making it a marginally LSCS model. While these models are promising, further studies are essential to determine their capacity to reliably replace spaceflight experiments, particularly in mimicking the intricate interplay of stressors experienced in space.

In ground-based models, thymic involution is primarily associated with loss of double-positive (CD4^+^CD8^+^) thymocytes, a sensitive subset that often serves as an early indicator of stress-induced thymic damage (171–177). Thymic involution in these models is typically observed following short-term exposures to stressors such as monoenergetic radiation beams or brief hindlimb unloading. Such changes are often driven by overstimulation of the hypothalamic-pituitary-adrenal (HPA) axis, resulting in elevated corticosterone or cortisol levels. However, space missions are expected to impose prolonged stressors, necessitating models that investigate the effects of extended exposures. Notably, ground-based experiments involving longer durations, such as extended hindlimb unloading or sophisticated galactic cosmic ray (GCR) simulations, reveal distinct thymic alterations. Beyond thymocyte loss, these exposures significantly affect the thymic stroma, particularly thymic epithelial cells (TECs) (25), which are critical for maintaining thymic architecture and supporting thymocyte development and selection (178–184). This shift underscores that long-term stressors may more profoundly impair the thymus by targeting its regenerative infrastructure rather than inducing acute thymocyte depletion. Accordingly, future countermeasures should prioritize the preservation and regeneration of the thymic stroma, especially TECs, to ensure the recovery and sustained functionality of the thymus during prolonged spaceflight.

Thymic involution during spaceflight poses both immediate and long-term risks to astronaut health (25). Interestingly, the recovery of thymic function shortly after returning to Earth was shown in astronauts (82), highlighting the organ’s regenerative capacity and intrinsic plasticity. However, during extended missions in deep space, the thymus may face sustained functional compromise. Prolonged thymic involution could lead to a diminished T-cell receptor repertoire, impaired immune surveillance, and weakened systemic immunity (173, 185–187). These effects may heighten susceptibility to infections, including reactivation of latent viruses, and potentially increase the long-term risk of cancer or other immunological diseases (25, 188, 189). While limited epidemiological data do not currently suggest a higher cancer incidence among astronauts compared to the general population (190–192), further monitoring and research are critical to comprehensively assess these risks. The thymus plays a particularly vital role in children, where it establishes a diverse and robust T-cell receptor repertoire (175). In adults, while peripheral expansion of existing T-cell clones predominates, the thymus remains essential for generating new T-cell receptor diversity, enhancing immune adaptability to novel pathogens, and even a subtle but prolonged thymic decline could potentially have significant consequences (193). For instance, a large study found that patients undergoing thymectomy as part of chest surgery had significantly reduced overall survival compared to those undergoing similar surgeries without thymectomy, underscoring critical role of the thymus in adult immunity (194). Therefore, to safeguard astronaut health in prolonged space exploration, it is imperative to prevent or mitigate spaceflight-induced thymic involution (52).

Despite the progress and advances using ground-based models to simulate spaceflight-induced thymic involution, critical questions still remain. How do specific thymic subsets, such as medullary thymic epithelial cells and early thymic progenitors, respond to prolonged low-dose, mixed-field galactic cosmic radiation (GCR)? What specific molecular pathways disrupted by GCR differentiate its effects from other forms of ionizing radiation, and which is the molecular basis for such differences? Moreover, the interplay between corticosterone-driven apoptosis and apoptosis-independent mechanisms affecting thymocytes and thymic architecture is still unclear. Moreover, the fidelity of these models raises questions: to what extent do fluid shifts and psychological stress in the HU model skew results away from microgravity’s true impact on the thymus? How can these variables be isolated? Future studies should integrate advanced molecular imaging and single-cell technologies, to gain an in-depth understanding of the mechanistic underpinnings behind spaceflight-induced thymic involution, and to support the development of rationalized countermeasures for astronaut health in long-term space missions.
